# Curcuminoid submicron particle ameliorates cognitive deficits and decreases amyloid pathology in Alzheimer’s disease mouse model

**DOI:** 10.18632/oncotarget.24369

**Published:** 2018-01-31

**Authors:** Yi-Heng Tai, Yu-Yi Lin, Kai-Chen Wang, Chao-Lin Chang, Ru-Yin Chen, Chia-Chu Wu, Irene H. Cheng

**Affiliations:** ^1^ Institute of Brain Science, National Yang-Ming University, Taipei, Taiwan; ^2^ Department of Neurology, Cheng-Hsin General Hospital, Taipei, Taiwan; ^3^ Food Industry Research and Development Institute, Hsinchu, Taiwan; ^4^ Brain Research Center, National Yang-Ming University, Taipei, Taiwan

**Keywords:** Alzheimer’s disease, curcuminoid submicron particle, APP transgenic mouse, amyloid, curcumin

## Abstract

Alzheimer's disease (AD) is the most prevalent neurodegenerative disorder and is triggered via abnormal accumulation of amyloid-β peptide (Aβ). Aggregated Aβ is responsible for disrupting calcium homeostasis, inducing neuroinflammation, and promoting neurodegeneration. In this study, we generated curcuminoid submicron particle (CSP), which reduce the average size to ~60 nm in diameter. CSP had elevated the bioavailability *in vivo* and better neuroprotective effect against oligomeric Aβ than un-nanosized curcuminoids *in vitro*. Two months of CSP consumption reversed spatial memory deficits and the loss of a calcium binding protein calbindin-D_28k_ in the hippocampus of AD mouse model. In addition, CSP consumption lowered amyloid plaques and astrogliosis *in vivo* and enhanced microglial Aβ phagocytosis *in vitro*, implying that the beneficial effects of CSP also mediated via modulating neuroinflammation and enhancing amyloid clearance. Taken together, our study demonstrated the protective effects of CSP toward ameliorating the memory impairment and pathological deficits in AD mouse model.

## INTRODUCTION

Alzheimer's disease (AD) is the most common form of dementia affecting more than 46 million patients worldwide. Abnormal accumulation of extracellular amyloid-β peptide (Aβ) into amyloid plaques in the brain is one of the pathological hallmarks of AD. Aβ is produced through the sequential proteolysis processing of amyloid precursor protein (APP) by β- and γ-secretases. Among different length of Aβ, Aβ40 is the most abundant species and accounts for 90% of total Aβ in the brain. However, Aβ42 is more aggregation-prone and more neurotoxic than other Aβ species, and thus play the major pathogenic role in AD [1, 2]. Overexpression of Aβ deteriorates the cognitive function; on the contrary, reduced levels of Aβ are often associated with alleviating the cognitive deficits [3]. Impaired clearance of Aβ is one of the major the factors that result in the cognitive dysfunction in sporadic AD patients [4].

Neuroinflammation triggered by the activation of astrocyte and microglia plays a central role in the pathogenesis of AD [5, 6]. Astrocytes are a key regulator of neuroinflammation and important for maintaining neuronal functions. Extensive proliferation of astrocytes induced by Aβ with a reactive phenotype and abnormal regulation leads to cognitive decline in AD [7]. Microglia, the primary immune cells of the brain, play an important role in maintaining neuronal function and protecting the brain from insults. The activation of microglia has both beneficial and detrimental roles in AD. Activated microglia could be classified into two phenotypes: M1 inflammatory microglia and M2 anti-inflammatory microglia [8]. M1 phenotype microglia can be triggered by Aβ to produce pro-inflammatory cytokines, which drive downstream cytokine storm to induce cytotoxicity [9]. In contrast, M2 phenotype microglia play protective roles against Aβ -induced damages [10–12].

Curcuminoid is a group of natural polyphenol consisted of diarylheptanoid compounds derived from the rhizomes of *Curcuma Longa*, such as curcumin and demethoxycurcumin. Epidemiological studies suggested that curcuminoid consumption is highly associated with the lower prevalence of AD in India [13, 14]. In addition, curcumin could inhibit neuroinflammation and reduce amyloid deposition in AD mouse model [15–17]. However, curcumin intake failed to reduce the amyloid levels in AD patients in the clinical trial [18]. The major limitation using curcuminoid as a treatment/protective agent is its low bioavailability, which is caused by its poor water solubility and low absorption rate in the gastrointestinal tract [19, 20]. Several approaches have been applied to overcome this problem, including structural modifications, pharmaceutical adjuvants, liposomes, and nanoparticles [19, 21, 22]. However, none of them significantly improve the spatial memory deficits in AD mouse model. Nanoparticle technology has emerged as a promising access to enhance bioavailability of lipophilic molecules such as curcumin [22–24]. The advantage of this nanoparticle technology is to reduce the average size of curcuminoid without structure modification that may alter curcuminoid nature properties.

In this study, we produced curcuminoid submicron particle (CSP) to average size around 60 nm in diameter and investigate the neuroprotective effects of CSP *in vitro* and *in vivo*. We found that CSP had higher bioavailability, improved spatial learning and memory, and reduced amyloid pathology in APP transgenic mouse. Furthermore, CSP could inhibit neuroinflammation and promote phagocytosis to clear Aβ. Our study suggested a potential use of CSP for future AD intervention.

## RESULTS

### CSP had better protective effect against oligomeric Aβ *in vitro*

**Figure 1:**
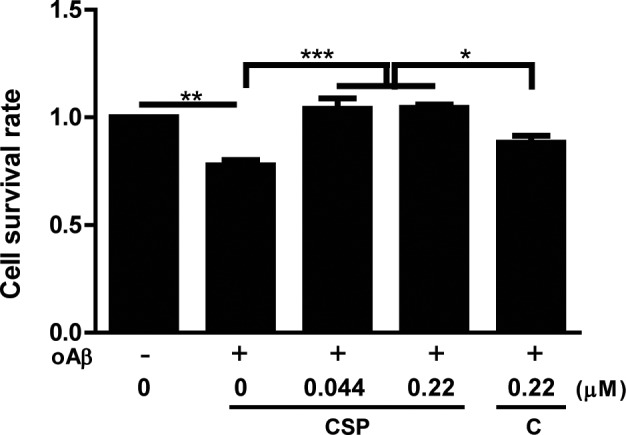
CSP had better protective effect against oligomeric Aβ (oAβ) than un-nanosized curcuminoid *in vitro* SH-SY5Y cells were pre-treated with 0, 0.044 or 0.22 μM curcuminoid submicron particle (CSP) or 0.22 μM un-nanosized curcuminoid (C) for 4 hours and then co-treated with 5 μM oAβ for 48 hours. The cell survival rate was determined by MTT assay in 3 independent experiments (N = 8 per experiment). Results were analyzed by one-way ANOVA. ^***^, P < 0.001; ^**^, P < 0.01; ^*^, P < 0.05. The survival rate of SH-SY5Y cells treated with vesicle control was set as 1.

To compare the neuroprotective effect, oligomeric Aβ (oAβ) treated SH-SY5Y human neuroblastoma cells were co-incubated with 5 μM curcuminoid submicron particle (CSP) or un-nanosized curcuminoids (C) for 48 hours, and their viability was determined by MTT assay. The survival rate in oAβ treated cells was significantly decreased compared with non-treated cells. Co-incubation with 0.044 and 0.22 μM CSP or un-nanosized curcuminoids significantly reversed oAβ-induced neuronal death. In particular, 0.22 μM CSP treated cells had significantly higher survival rate than un-nanosized curcuminoids under Aβ stress (Figure 1). Because CSP had better neuroprotective effect against Aβ *in vitro*, we further tested the potential use of it to prevent neurodegeneration *in vivo*.

### Genotoxicity and biosafety of CSP

Before applying to AD animal model, we first examined the biosafety of CSP after oral consumption. The acute genotoxicity of CSP was determined using micronucleus assay. ICR male mice were administrated with 0.03, 0.3, and 3 g/kg of CSP, vesicle (negative control), or cyclophosphamide (positive control). The percentage of micronucleated erythrocytes in plasma was used as an indicator of chemical-induced genotoxicity. The percentage of micronucleated erythrocytes in CSP treated groups had no significant difference in comparison with vesicle control group, suggesting that CSP did not induce significant genomic instability and toxicity (Supplementary Table 1).

**Figure 2:**
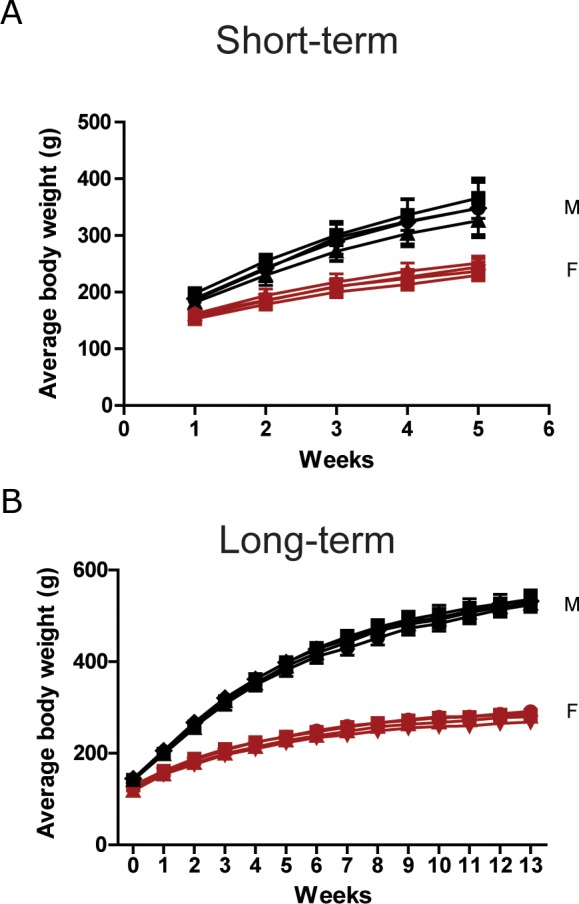
Body weight of SD rats in the oral toxicity study of CSP Male (black) and female (red) SD rats received 0 (●), 0.1 (▄), 0.5 (▲), and 1 (▼) g/kg/day of CSP **(A)** for 28 days and **(B)** 90 days. N = 6 mice/group in 28-day test. N = 8 mice/group in 90-day test. Results were analyzed by one-way ANOVA.

The biosafety of CSP was determined by administration of 0, 0.1, 0.5, and 1.0 g/kg /day of CSP to male and female Sprague-Dawley (SD) rats for continuous 28 days (short-term) and 90 days (long-term). All groups had no apparent adverse effects and recorded death (Supplementary Table 2). Furthermore, there were no significant changes in average body weight (Figure 2A-2B), and organ weights (Supplementary Table 3-4) among these groups.

### Bioavailability of CSP

Low bioavailability is one of the major hinders of curcuminoid to be applied as a therapeutic agent [18]. To assess the bioavailability of CSP, ICR mice were gavaged with low dose (0.2 g/kg) or high dose (2.5 g/kg) of un-nanosized curcuminoids (C) or curcuminoid submicron particle (CSP), and their plasma were collected at 0, 15, 30, 45, 60, 120, and 300 minutes after gavaging. The level of un-nanosized curcuminoids or CSP in plasma was determined by high-pressure liquid chromatography (HPLC). The pharmacokinetic analysis indicated that the CSP had 35-folded higher absorbability than un-nanosized curcuminoids in high dose group (Table 1), illustrating that reducing the particle size could effectively improve the pharmacokinetic properties of curcuminoids.

**Table 1:** Pharmacokinetic analysis of un-nanosized curcuminoid (C) and curcuminoid submicron particle (CSP)

Treatment	Dosage (g/kg)	C_max_ (μg/ml)	T_max_ (min)	AUC (μg/ml∙min)
C	0.2	0.47±0.33	45±11	36±12
CSP		5.96±0.72	75±42	1,250±56
Ratio (CSP/C)		12.62	-	35.16
C	2.5	1.83±0.19	48±7	276±21
CSP		12.70±1.01	55±7	1,884±57
Ratio (CSP/C)		6.96	-	6.82

C_max_: peak concentration. T_max_: time to reach peak concentration. AUC: area under curve (plasma concentration-time). C: curcuminoid. CSP: curcuminoid submicron particle. Quantitative data are listed as the mean ± standard deviation.

### CSP ameliorated spatial learning and memory deficit of APP mice

**Figure 3:**
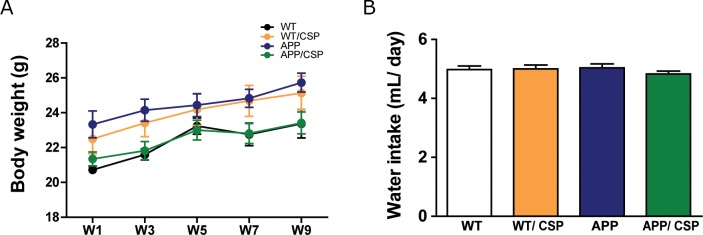
Body weight and water intake of WT and APP mice during CSP treatment APP and wildtype littermate control (WT) mice received 0 or 0.75 mg/mL/day of CSP for 2 months. **(A)** Body weight and **(B)** water intake were recorded weekly during CSP administration. N = 12-17 mice/group. Results were analyzed by one-way ANOVA.

The neuroprotective effects of CSP *in vivo* were examined using APP transgenic mouse (line J20), which generates high level of Aβ [25] and has age-dependent functional and pathological deficits onset at 4 months of age [26, 27]. APP mice and wild-type (WT) littermate controls were administered with CSP or vesicle control at 0.75 mg/ml in drinking water starting at 3 months of age for two months. The average amount of CSP consumption was 37.5 mg/kg/day. These mice had no significant difference in average body weight (Figure 3A) and water intake (Figure 3B) after 2 months treatment. AD-like functional and pathological deficits were examined in following four groups: wild-type fed with vesicle (WT), wild-type fed with CSP (WT/CSP), APP fed with vesicle, and APP fed with CSP (APP/CSP).

**Figure 4:**
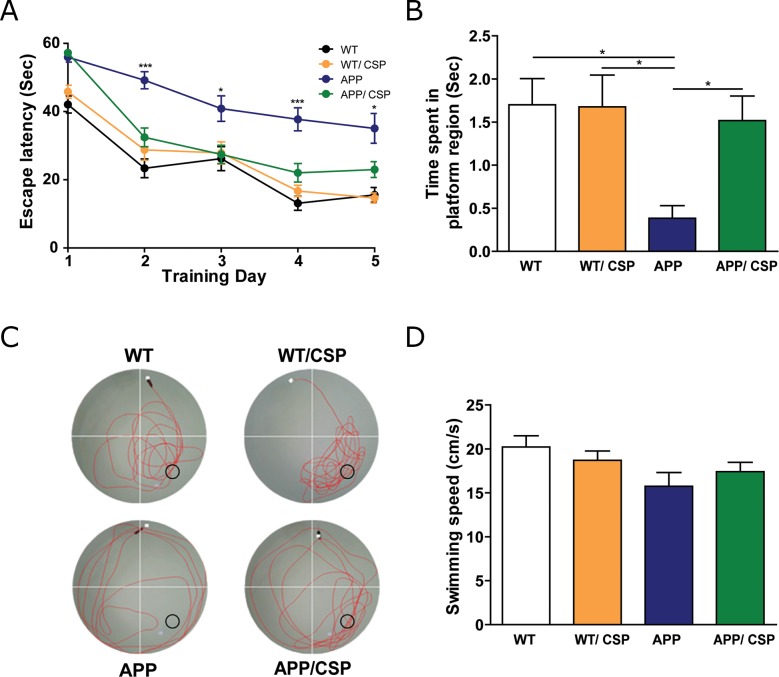
CSP ameliorated the spatial learning and memory of APP mice in the Morris water maze **(A)** In hidden platform test, APP/CSP mice had a lower escape latency than APP mice given the control diet. **(B)** In probe trial, APP/CSP mice spent more time in platform region than APP mice. **(C)** Representative traces of each group in the probe trial. ○ = platform location. **(D)** Swimming speed had no significant difference among all four groups. N = 12-17/group. Results were analyzed by one-way ANOVA.^*^, p < 0.05; ^***^, p < 0.001.

To elucidate whether CSP could improve cognitive impairment of APP mice [26, 28], the Morris water maze was adopted to evaluate spatial memory deficits the in these 4 groups of mice. In memory acquisition session, the escape latency to reach the hidden platform was longer in APP mice than WT mice. APP/CSP mice exhibited significantly shorter escape latency than APP mice in the last 4 days (Figure 4A). In probe trial for memory retention, APP mice spent significantly less time in the platform region than WT, and APP/CSP mice spent significantly longer time in the platform region than APP mice (Figure 4B-4C). There was no difference between WT and WT/CSP groups in all the tests. The swimming speeds among each group had no significant difference (Figure 4D), suggesting that the reverse of memory deficits in APP/CSP mice was not due the impairment in motor function. Our results demonstrated that CSP significantly improved both memory acquisition and the memory retention deficits in APP mice.

### CSP did not alter anxiety and locomotor behaviors of APP mice

**Figure 5:**
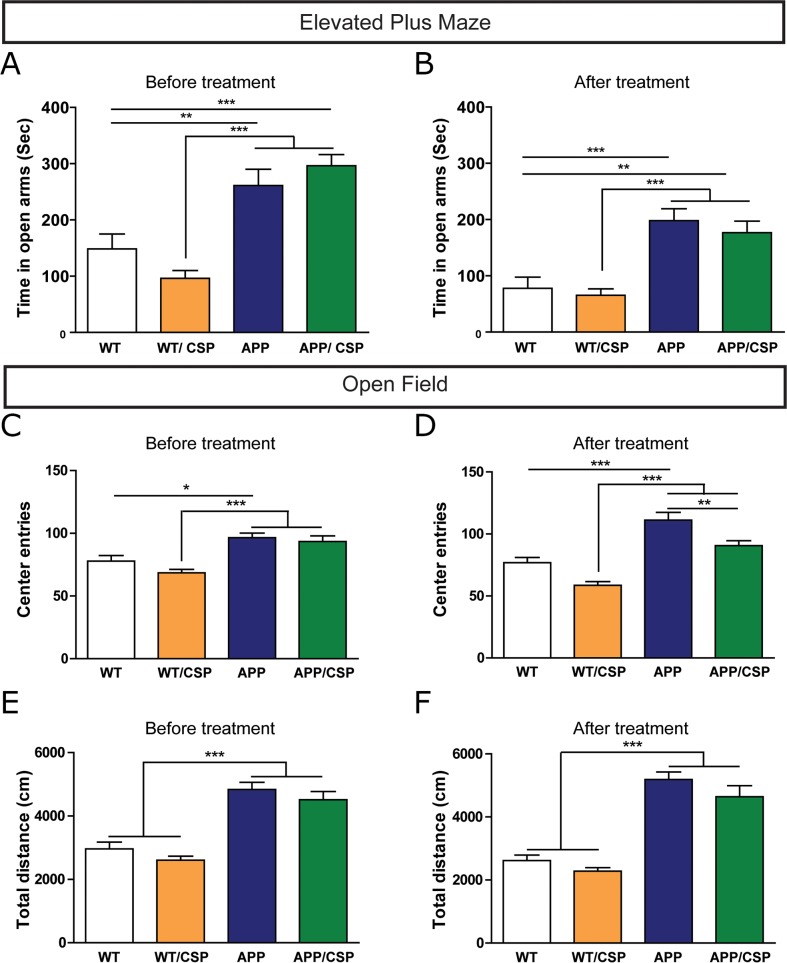
Effect of CSP on anxiety-related behavior and locomotor activities of APP mice **(A-B)** In elevated plus maze, anxiety behavior of these mice was measured by time in open arms before (A) and after (B) CSP treatment. **(C-F)** In open field, anxiety was measured by the number of center entries (C, D) and locomotor activity was measured by total distance traveled (e, f) before and after CSP consumption. N = 12-17 mice/group. Results were analyzed by two-way ANOVA. ^*^, p < 0.05; ^**^, p < 0.01; ^***^, p < 0.001.

Before and after CSP consumption, we used the elevated plus maze to screen for anxiety-related behavior, and the open field test to monitor anxiety and locomotor activity. Compared with WT mice, APP mice spent more time in the open arm of the elevated plus maze (Figure 5A-5B), and traveled a longer distance and explored in the center region more frequently in the open field (Figure 5C-5F), consistent with previous findings [12, 27, 28]. In the elevated plus maze, 2 months of CSP treatment did not significantly reverse the higher open arm time in APP mice (Figure 5A-5B). Nevertheless, in the open field, 2 months of CSP treatment reduced the number of center entries (Figure 5C-5D) but did not alter the total distance moved in APP mice (Figure 5E-5F). Compared with WT mice, WT/CSP mice had no change in anxiety-related behavioral or locomotor activity. In summary, CSP did not influence locomotor activity and only partially reversed anxiety-related behavioral in APP mice.

### CSP reversed the calbindin-D_28K_ level in the hippocampus of APP mice

**Figure 6:**
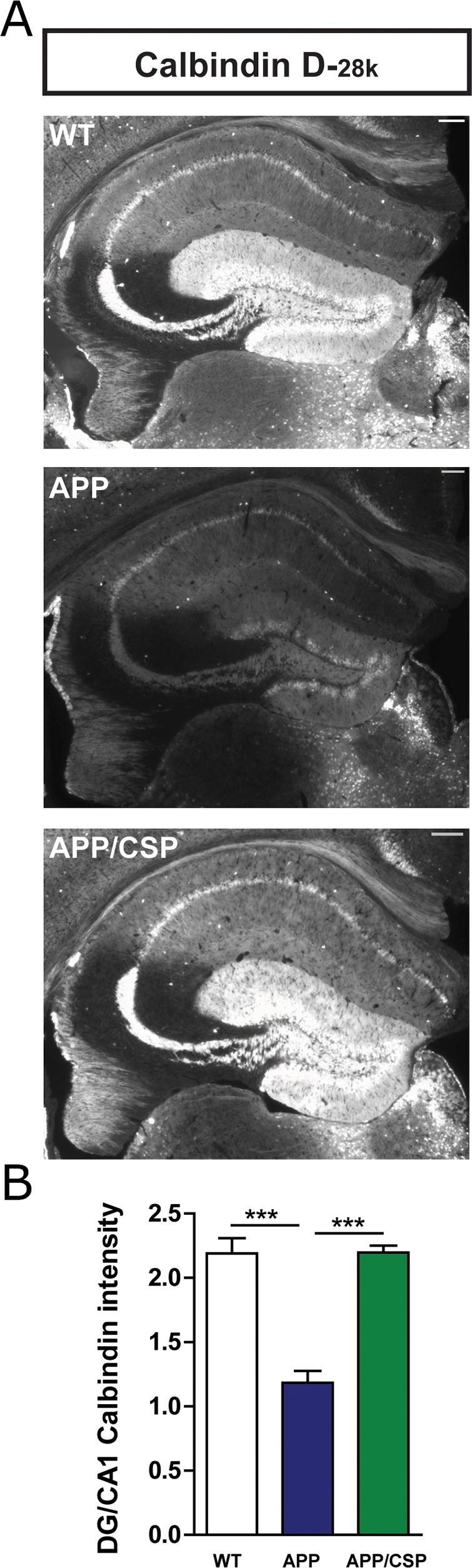
CSP reversed the calbindin-D_28K_ level in the hippocampus of APP mice **(A)** Representative calbindin-D_28K_ images in the hippocampus of WT, APP, APP/CSP mice. **(B)** Normalized intensity of calbindin-D_28K_ in the dentate gyrus of the hippocampus in each group. N = 6 mice/group, 8-10 brain slices per mouse. Results were analyzed by one-way ANOVA. ^***^, p < 0.001. Scale bar = 200 μm.

Memory deficits in APP mice are correlated with the reduced levels of a calcium-binding protein calbindin-D_28K_ and calcium dysregulation in the dentate gyrus [26, 29]. Therefore, the expression of calbindin-D_28K_ was used to as a marker to examine calcium homeostasis in our mice. We found that the level of calbindin-D_28K_ was significantly reduced in APP mice compared to WT mice, but this reduction can be alleviated by CSP consumption in APP mice (Figure 6A-6D), implying that memory decline in APP mice rescued by CSP could be associated with the revered levels of calbindin-D_28K_ in the dentate gyrus.

### CSP decreased the amyloid level and astrogliosis in the hippocampus of APP mice

**Figure 7:**
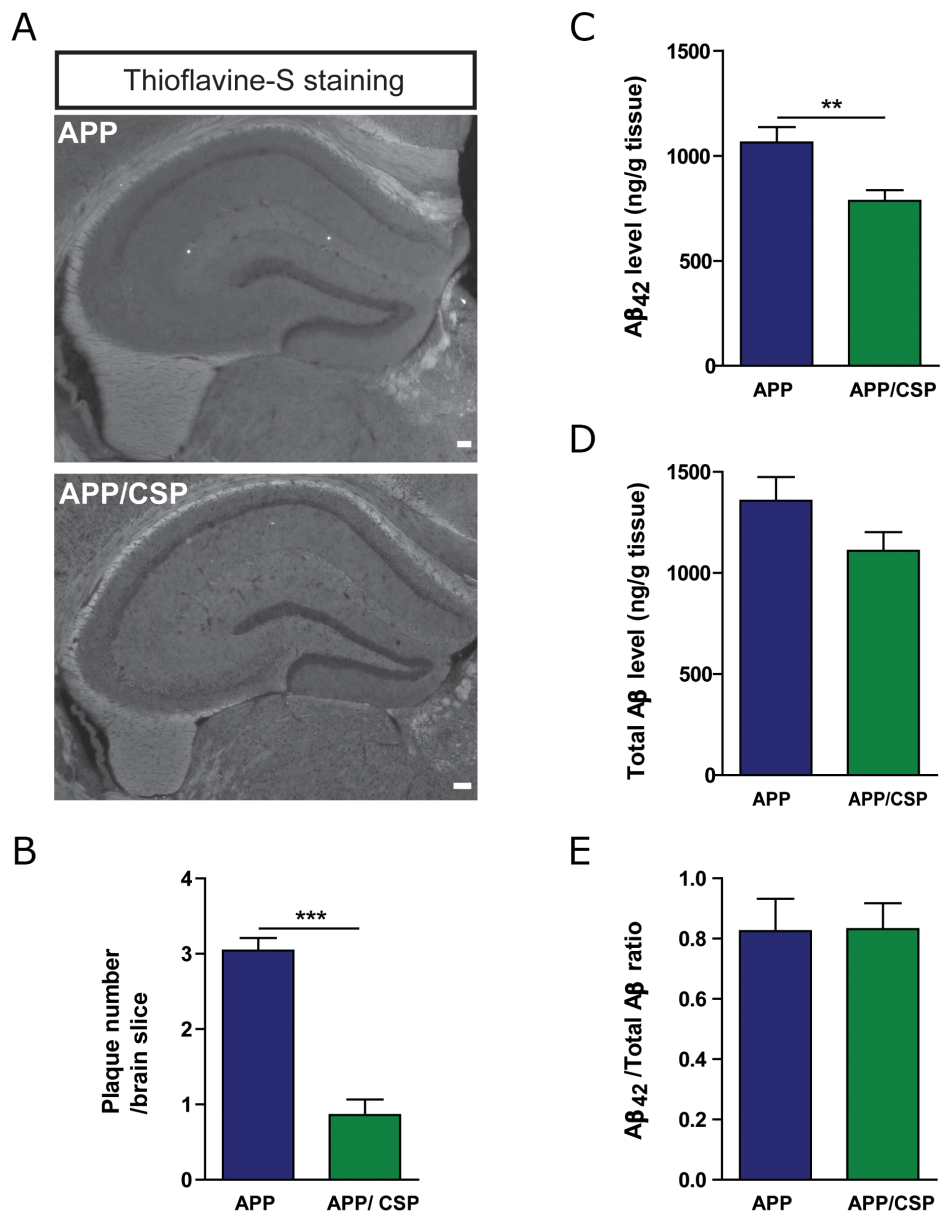
CSP decreased the amyloid deposition in the hippocampus of APP mice **(A)** Representative images of β-sheet amyloid plaques in the hippocampus of APP and APP/CSP mice stained by Thioflavin-S. Scale bar = 200 μm. **(B)** Quantitative analysis of the number of the plaques in the hippocampus. N = 6 mice/group, 6-10 slices per mouse. **(C-D)** The levels of Aβ42 (C) and total Aβ (D) in the hippocampal lysate were determined by ELISA. **(E)** Aβ42/total Aβ were unchanged in APP mice treated with CSP. N = 13-17 mice/group. Results were analyzed by *t* test. ^**^, p < 0.01; ^***^, p < 0.001 versus APP mice.

Aβ deposition is one of the most important pathological hallmarks of AD. Among different length of Aβ peptides, Aβ42 is more aggregation-prone and more neurotoxic than other Aβ species [3]. To investigate whether CSP alters Aβ level in APP mice, we monitored the appearance of amyloid plaques with thioflavin-S staining and the level of Aβ with enzyme-linked immunosorbent assay (ELISA). The number of amyloid plaques (Figure 7A-7B) and the level of Aβ42 (Figure 7C) were significantly decreased in the hippocampus of APP/CSP mice compared to those of APP mice. However, there were no significant reductions in total Aβ level (Figure 7D) and Aβ42 to total Aβ ratio (Figure 7E) between APP/CSP mice and APP mice. These results indicated that CSP could effectively inhibit the amyloid and neurotoxic Aβ 42 levels in AD.

**Figure 8:**
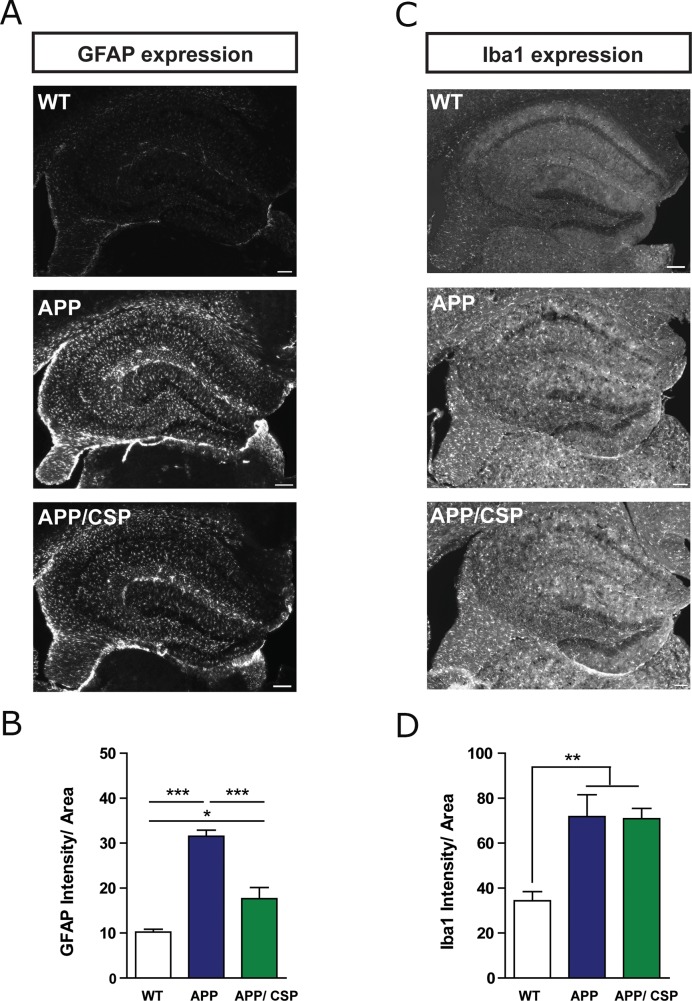
CSP inhibited astrocyte activation in the hippocampus of APP mice **(A)** Representative images of GFAP^+^ astrocyte activation in the hippocampus of WT, APP and APP/CSP mice. **(B)** Normalized GFAP intensity in the hippocampus in each group. **(C)** Representative images of Iba1^+^ microglia in the hippocampus of WT, APP and APP/CSP mice. **(D)** The normalized Iba1 intensity in the hippocampus in each group. N = 6 mice/group, 8-10 brain slices per mouse. Results were analyzed by one-way ANOVA. ^*^, p < 0.05; ^**^, p < 0.01; ^***^, p < 0.001. Scale bar = 200 μm.

In addition to amyloid pathology, the inflammatory response in these mice was measured by the activation of astrocyte or microglia. The immunoreactive signals of glial fibrillary acidic protein (GFAP) and ionized calcium-binding adapter molecule 1 (Iba1) were used as astrocyte and microglial markers [30]. We found that APP mice had higher GFAP and Iba1 intensity than WT mice. Consumption of CSP reduced the intensity of GFAP positive astrocyte in the hippocampus of APP/CSP mice (Figure 8A-8B). CSP did not alter the intensity of Iba1-positive microglia in the hippocampus of APP mice (Figure 8C-8D). Taken together, CSP could mitigate amyloid pathology and inflammatory reaction in APP mice.

### CSP promoted the microglial phagocytosis and Aβ uptake in BV_2_ microglia

**Figure 9:**
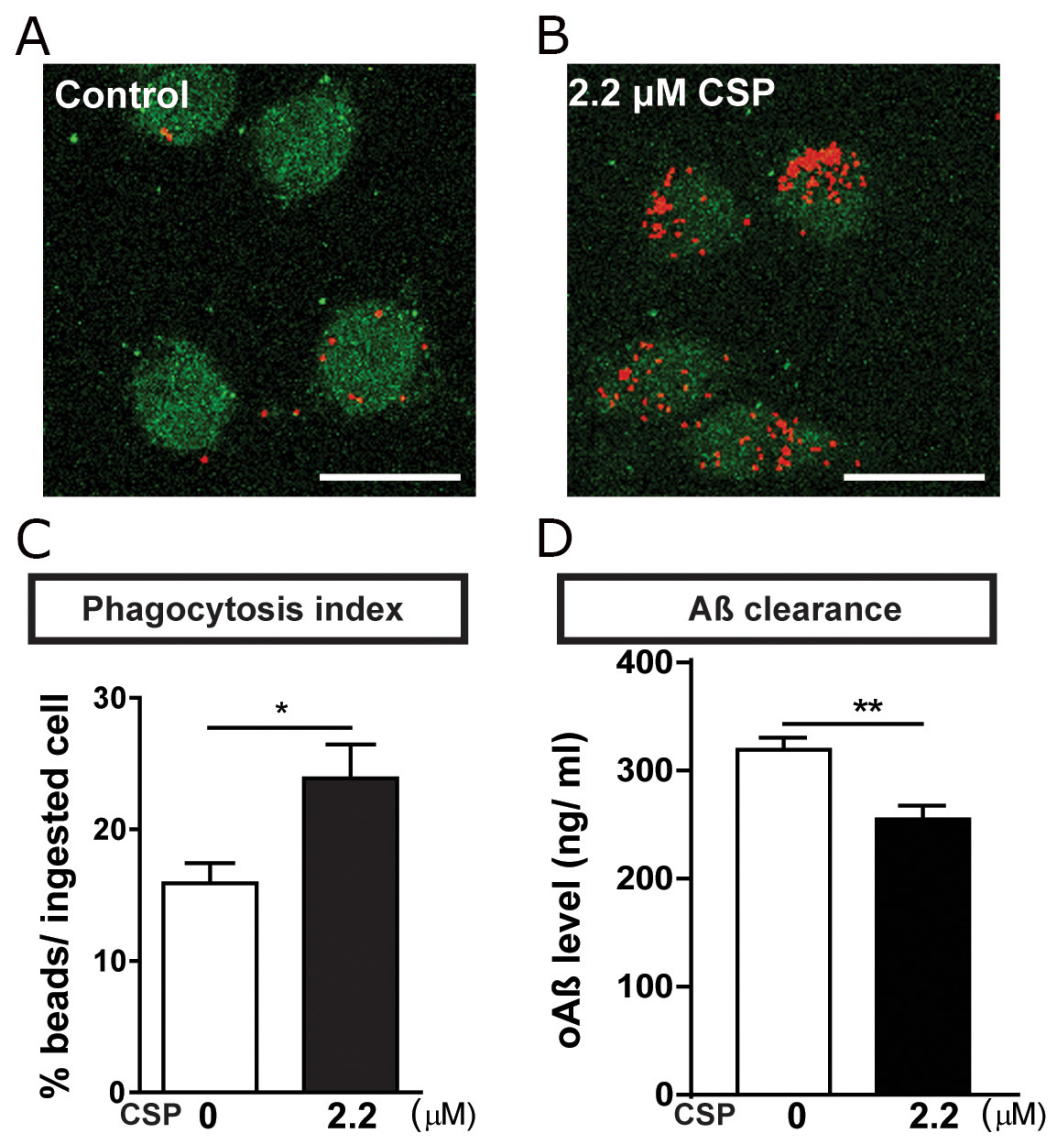
CSP increased phagocytosis of BV_2_ microglia **(A-B)** Representative images of ingested microspheres (red) in the BV_2_ cells (green) treated with 0 or 2.2 μM CSP for 1 hr. Scale bar = 25 μm. **(C)** CSP significantly increased the phagocytosis index. N = 11,480 cells in control group; N = 18,199 cells in CSP treated group. **(D)** BV_2_ cells were treated with 1 μM oAβ and 0 or 2.2 μM CSP for 24 hrs. Levels of residual Aβ in the media were lower in the CSP treated group. Results were analyzed by *t* test. ^*^, p < 0.05; ^**^, p < 0.01.

Microglia activation could promote phagocytosis to clear Aβ. Although CSP did not reduce activated microglia, CSP decreased the number of plaques in the hippocampus of APP mice. Therefore, we further identified whether CSP could affect the Aβ clearance through enhancing the microglial phagocytosis [3]. To address this question, the BV_2_ microglial cell was treated with 0 or 2.2 μM CSP for 1 hour and followed by adding 0.001% fluorescent microspheres beads (Figure 9A-9B). We found that CSP treated cells had significantly higher percentage of phagocytosed cells (Figure 9C). Furthermore, after co-incubating oAβ with or without CSP, the Aβ level in the medium of Aβ+CSP-treated microglia was significantly lower than Aβ only microglia (Figure 9D). These results demonstrated that CSP enhances the Aβ -clearance ability of microglia, thereby ameliorating Aβ-induced neurodegeneration.

**Figure 10:**
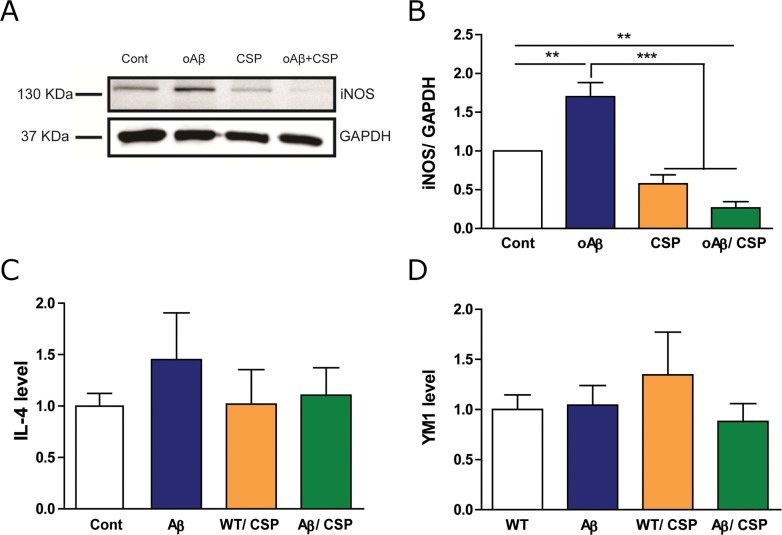
CSP reduced the M1 but did not change the M2 type of microglia under oAβ stress BV_2_ microglia was treated with 5 μM oAβ and 2.2 μM CSP for 24 hours. **(A)** Representative immunoblot images for pro-inflammatory M1 type microglia marker iNOS in BV_2_ cells. **(B)** The level of iNOS in oAβ+CSP treated microglia was significantly lower than oAβ treated microglia. **(C-D)** Expression of anti-inflammatory M2 type microglia markers YM1 and IL-4 RNA had no significant difference among all groups. Results were analyzed by one-way ANOVA. ^**^, p < 0.01; ^***^, p < 0.001.

We further determined whether CSP could modulate the ratio of M1/M2 phenotype of microglia. The exposure of BV2 to 5 μM oAβ could significantly increase the inducible nitric oxide synthase (iNOS), which is one of the direct consequences of an inflammatory process and commonly uses as a marker for M1 microglia [31, 32]. The iNOS level was decreased in BV_2_ microglia co-treated with 2.2 μM CSP and oAβ (Figure 10A-10B). However, there was no change in M2 anti-inflammatory markers YM1 and IL-4 (Figure 10C-10D). These results suggested that CSP may decrease neuroinflammation but did not alter M1/M2 microglia phenotypes.

### CSP did not have anti-aggregation effect on Aβ

**Figure 11:**
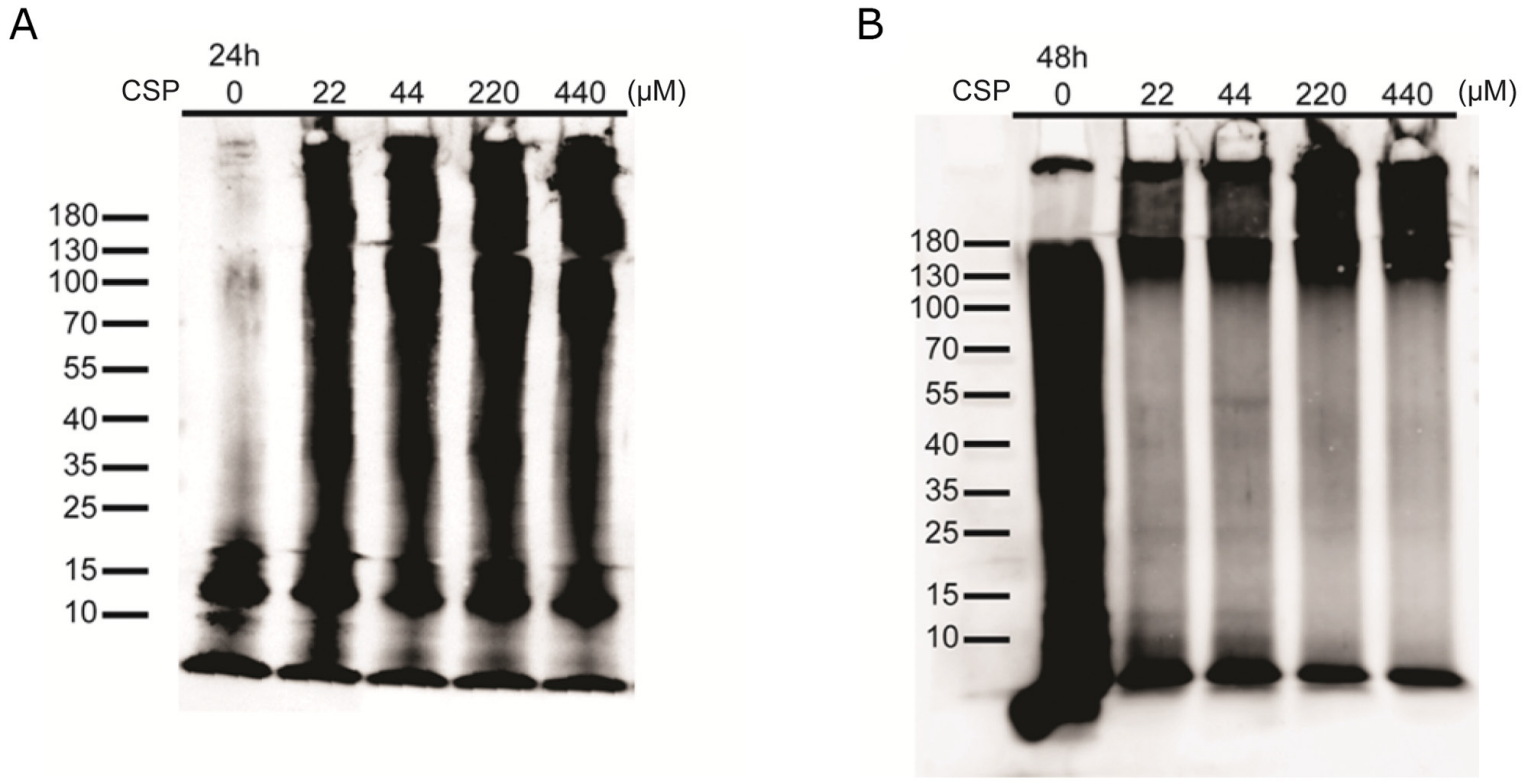
CSP did not inhibit Aβ aggregation *in vitro* Representative images of Aβ aggregation states in the presence of CSP. 5μM monomeric Aβ were co-incubated with 0, 22, 44, 220, and 440 μM CSPs for **(A)** 24 hours and **(B)** 48 hours. The size distribution of aggregated Aβ was immediately examined by western blot.

The reduced Aβ level in APP/CSP mice may also due to the inhibition of Aβ aggregation by CSP. Curcumin has been reported to reduce the amounts of higher molecular Aβ aggregates [16]. To monitor the effect of CSP on Aβ aggregation, 5 μM monomeric Aβ was incubated with 0, 22, 44, 220, 440 μM CSP for 24 and 48 hours and subjected to immunoblot analysis. We found that Aβ aggregates into high molecular weight assemblies (>180 kDa) faster in the presence of CSP, indicating that the reduction of Aβ deposition in the APP/CSP mice is not due to the blockage of Aβ aggregation (Figure 11).

## DISCUSSION

This study demonstrated that the nano-sized curcuminoid, CSP, had higher bioavailability and better neuroprotective effects than un-nanosized curcuminoid. CSP treatment significantly ameliorated the cognitive function, reduced the amyloid deposition, decreased astrogliosis, reversed calbindin-D_28k_ and enhanced microglial phagocytosis. These findings indicate that CSP has potential to be applied as a prevention agent for AD. Curcumin and curcuminoid do not induce apparent adverse effects up to 8,000 mg/day in healthy adults [33–35]. We demonstrated that both short term and long term CSP consumption is safe under 1000 mg/kg/day. In this study, the amount of CSP consumption for the mouse was 187.5 mg/kg/day, which is approximately equated to 15.2 mg/kg/day for human [36]. For a 60 kg adult, daily intake of CSP needs to be 912 mg to reach the similar neuroprotective effect, which is within the safe range for curcuminoid [37–39].

Multiple approaches have been applied to increase the bioavailability and enhance the neuroprotective effect of curcumin or curcuminoid. The two most common approaches were structural modification and size reduction [22–24]. After oral gavage, the plasma concentration of CSP was 6-35 times higher than un-nanosized curcuminoid, which is similar or even better than the bioavailability of curcumin modified by other approaches [22–24]. Whether curcumin could reverse the memory-related deficits in AD mouse models have diverse results. In other curcumin treated AD mouse model, although curcumin could reverse working memory deficits in Y-maze test [38, 40], it did not significantly improve the memory retention deficits in the probe trial of the Morris water maze test, which is more related to the condition in AD patients [37, 39, 41]. The PLGA nanoparticles modified curcumin could only attenuate memory deficits when co-delivering with Aβ generation inhibitor through intraperitoneal injection to AD mouse model [42]. In our study, APP/CSP mice performed significantly better in both hidden platform and probe trial tests, suggesting that the improvement in both memory acquisition and retention of AD mouse model. Our results indicated that the therapeutic efficacy of orally administered CSP was greatly increased in comparison with previous studies.

Aβ -induced neuroinflammation is mostly mediated through CNS-resident cells, including astrocyte and microglia, rather than invading immune cells [43]. Reactive astrocytes cause disruptions in synaptic connectivity, imbalance of neurotransmitter homeostasis, and neurodegeneration in AD [44, 45]. Furthermore, the degree of astrogliosis is correlated with cognitive decline in the brain of AD patients [46]. In our study, CSP could inhibit reactive astrocyte activation in APP mice, suggesting that CSP could reduce astrocyte-mediated neuroinflammation. On the other hand, although CSP consumption did not alter the intensity of Iba1^+^ microglia in APP mice, CSP treatment enhanced phagocytic percentage in BV_2_ microglia cells. Therefore, the reduction of amyloid plaques in the hippocampus of APP mice might be mediated by CSP through the microglial engulfment. Dysfunction of microglial phagocytosis in AD patients has been linked to the disrupted clearance of Aβ, and the enhanced memory impairment [47, 48]. A curcuminoid compound (bisdemethoxycurcumin) has been shown to increase the Aβ phagocytosis in the brains of AD patients [49]. Our results provided consistent evidence that CSP enhances microglial phagocytosis.

Calcium-binding proteins could regulate calcium homeostasis and protect neuron against calcium-mediated neurotoxicity [50]. The disruption of calcium-binding proteins signaling impairs the synaptic function [51]. In the hippocampus, calbindin-D_28k_ containing neurons play roles in memory formation and long-term potentiation [52]. The level of calbindin-D_28k_ is highly correlated with memory retention deficits of APP mice [26]. In our study, the reduced expression of calbindin-D_28k_ in APP mice was reversed after CSP consumption, which provided the first indication linking the effects of curcuminoid to the levels of calbindin-D_28k_ in AD model.

In summary, this study demonstrated the beneficial effects of CSP on spatial memory deficits and pathological changes in APP mice. CSP can be easily administered to animal model in drinking water as a stable suspension without noticeable adverse effects. Altogether, CSP could be a potential food supplement for long-term treatment of AD.

## MATERIALS AND METHODS

### Animals

Short-term (28-day) and long-term (90-day) biosafety tests were performed on male and female SD rats. Micronucleus assay was performed on ICR mice. AD mouse model used in this study is APP transgenic mice (line J20) carrying the human APP minigene with the Swedish (K670N/M671L) and Indiana (V717F) familial mutations. Animals were housed in a specific pathogen-free facility with a light/dark cycle of 12 hours light and 12 hours dark. Food and water for mice were provided ad libitum. Drinking water with 0.75 mg/mL CSP was administrated for mice from 3 to 5 months of ages. The open field and elevated plus maze were performed before and after treatment, and the Morris water maze was carried out after 2 months of CSP consumption. Mice were sacrificed with transcardial perfusion with 0.9% NaCl 2 days after behavioral tests. One hemibrain was drop-fixed in 4% paraformaldehyde for 48 h, and the other hemibrain immediately froze at −70°C. The study was approved by the Institutional Animal Care and Use Committee of National Yang-Ming University. All experimental procedures involving animals and their care were carried out in accordance with the Guide for the Care and Use of Laboratory Animals published by the United States National Institutes of Health.

### Preparation of aqueous dispersion with CSP

To prepare stabilizer for CSP, 2.5 g L-α-phosphatidylcholine (P7443, Sigma, USA) and 3.42 g sucrose esters (Gemfont Corporation, Taiwan) were sequentially incorporated into 400 mL water. The mixed stabilizer materials were stirred at 25°C, and 40 g curcuminoid powder with curcumin, demethoxycurcumin, and bisdemethoxycurcumin (Toong Yeuan, Taiwan) were added to form a 10 % curcuminoid aqueous solution. This non-homogenously mixed solution was subjected to a high-speed homogenization pretreatment from 4,000 to 6,000g for 10 minutes using a PRO250 homogenizer (PRO Scientific, USA). Next, a nano-grade wet grinder (Netzsch-Fein mahltechnik GmbH, Germany) carried on yttria-stabilized tetragonal zirconia for circulation milling with 0.2 mm beads for 180 minutes to obtain the aqueous dispersion. The average diameter of un-nanosized curcuminoid was 5140±178 nm, and the average diameter of CSP was 59±1 nm. Finally, the nanosized CSP composed of curcumin (83.56%), demethoxycurcumin (14.13%) and bisdemethoxycurcumin (2.31%) was obtained. Right before oral administration, CSP was diluted into the drinking water at concentration 0.75 mg/mL. The vesicle control in this study contain the same stabilizer and went through the same preparation process without adding curcuminoid.

### Bioavailability and biosafety analysis

For the pharmacokinetics analysis, ICR mice were administered by oral gavage with 0.2 g/kg or 2.5 g/kg of un-nanosized curcuminoid or CSP. At 15, 30, 45, 60, 120, and 300 minutes after gavage, plasma was collected and processed with sulfatase for 2 hours. The level of curcuminoid in plasma was determined by high-pressure liquid chromatography (HPLC).

For biosafety test, including genotoxic analysis, short-term and long-term tests were adopted to investigate the potentially harmful effects of CSP. Micronucleation assay was applied to identify the genotoxicity of CSP. ICR mice were administered orally with 0, 0.03, 0.3, 3.0 g/kg of CSP or 0.1 g/kg cyclophosphamide (positive control) for 48 hours. 200 μL plasma was incubated with 50 μL 100 U/mL sulfatase for 2 hours, and 120 μL processed sample was then fixed by frozen methanol (−80°C). 12 mL buffer (1.8g NaCl and 0.089g NaHCO_3_ in 200 mL sterile water, 4°C) was added to the fixed sample and centrifuged at 1,000 ×g for 5 minutes to remove supernatant. Cells were resuspended with 80 μL buffer (10 μL/mL CD71-FITC and 1 mg/mL RNAase) for 30 minutes at 4°C, and then 1 mL protease inhibitor (2.5 μg/mL) was added. The percentage micronucleated reticulocyte in their blood will be analyzed by Flow cytometric-Becton Dickinson FacsCalibur and Cell Quest.

In short-term and long-term safety tests, SD rats were administered with 0.1, 0.5 and 1.0 g/kg/day CSP, or sterile water, daily for 28 days and 90 days. The clinical condition, body weights, organ weights studies were performed after 28 days and 90 days.

### Morris water maze

The water maze consisted of a water pool (122 cm in diameter) containing opaque water and a platform (10 cm in diameter) submerged 1 cm below the water surface. The hidden platform test consisted of 10 sessions over 5 days, and each session comprised three 60-second trials with 15-minute inter-trial intervals. The platform location remained constant during the hidden platform sessions, and the entry points were changed semi-randomly between days. One day after the final day of hidden platform training section, a probe trial was conducted by removing the platform and allowing mice to explore in the pool for 1 minute. The time spent in platform region and swim speed were recorded and analyzed with an EthoVision video tracking system (Noldus, Wageningen, Netherlands).

### Elevated plus maze

Elevated plus maze consists of two open arms, two closed arms, and a center area. Mice were habituated in the testing room for 1 hour and then were placed individually at the center of the apparatus to explore for 10 minutes. The time spent and distances moved in each of the arms were recorded and analyzed with an EthoVision video tracking system.

### Open field

Mice were habituated in the testing room for 1 hour and were placed in an open arena (24.32 × 24.32 cm^2^) for 15 minutes. Two infrared photobeam sensor frames, each consisting of a 32 × 32 photobeam array, were used to detect movements in the horizontal and vertical plane (Version 2.0, TRU Scan Photobeam LINC, Coulbourn Instruments, PA, USA). The distance mice traveled and center entries were used as parameters to analyze the general activity and anxiety of mice.

### Enzyme-linked immunosorbent assay (ELISA)

Frozen hippocampi were homogenized in 5M guanidine/5mM Tris buffer (pH 8.0). The samples were diluted with 0.25 % casein blocking buffer containing 0.5 M guanidine and protease inhibitor mix (04693116001, Roche, Switzerland). Total Aβ and Aβ42 levels in the soluble fraction were analyzed with ELISA kits (27729 and 27711, INL, Germany) according to the manufacturer's instructions.

### Immunohistochemistry and thioflavin-S staining

Paraformaldehyde-fixed brains were sectioned coronally (at 20μm thickness) using a sliding microtome (CM1900; Leica, Germany). For immunohistochemistry (IHC), slices were blocked with phosphate-buffered saline (PBS) containing 10% fetal bovine serum (FBS) and 0.5% Triton X-100 for 1.5 hours, and incubated with anti-GFAP (Z0334; Dako Cytomation, Denmark), anti-Iba1 (019-19741; Wako, Japan), anti-YM1 (#01404, Stemcell Technologies, Canada) and anti-Calbindin-D_28K_ (CB38; Swant, Switzerland) antibodies at 4°C overnight. Slices were then incubated with Alexa Fluor 488-conjugated AffiniPure Goat anti-rabbit IgG secondary antibody (111–545–003; Jackson ImmunoResearch, USA) for 1.5 hours. For Thioflavin-S staining, slices were stained with 0.015% Thioflavin-S (T1892; Sigma, USA) for 15 minutes at room temperature. After mounting, slides were imaged using a Zeiss fluorescence microscope (Axio Observer A1; Zeiss, Germany).

### Cell culture

SH-SY5Y human neuroblastoma cell line was maintained in Minimum Essential Medium (MEM, 41500–034; Gibco, USA) plus F-12 nutrient mixture (21700–075; Gibco, USA) supplemented with 10% fetal bovine serum (FBS, SH3007; HyClone, USA), 0.11 g/L sodium pyruvate, and 1.69 g/L sodium bicarbonate. BV_2_ microglia cell line was maintained in Dulbecco's modified Eagle's medium (DMEM, 12100–046; Gibco, USA) supplemented with 10% FBS, 1% L-glutamine (GLL01; Caisson laboratory, USA), and 1.85 g/L sodium bicarbonate. Cell lines were grown at 37°C in a humidified 5% CO_2_ chamber.

### *In vitro* Aβ aggregation

HFIP treated Aβ (Ultra-pure Aβ42, HFIP, A1163-2; Kelowna) was dissolved with DMSO, and the Aβ-DMSO solution was then added into 10mM Tris/PBS buffer to form 100 uM Aβ solution. The pallet was further removed from Aβ solution by centrifugation (17000 ×g, 15 min, 4°C). The supernatant of 100 μM Aβ solution was then kept at 4°C for 24 h to form oligomer Aβ. The oligomeric Aβ was characterized by using immunoblotting (Figure 11A, CSP=0 μM).

To determine whether CSP could alter Aβ aggregation, 5 μM HFIP-Aβ was incubated with 0, 22, 44, 220, and 440 μM CSP for 24 and 48 hours, and their aggregation status was determined by immunoblotting.

### Immunoblotting

Cell lysates (10 μg of total protein) or aggregated Aβ peptide were separated via 10% Tris-glycine polyacrylamide gel electrophoresis, transferred to polyvinylidene difluoride (PVDF, IPVH00010; Millipore, Germany) membranes. Membranes were blocked in casein blocking buffer (B6429; Sigma Aldrich, USA) for 1 hours and probed with primary antibody for anti-iNOS (610328; BD Bioscience, USA), anti-GAPDH (G8795; Sigma Aldrich, USA) or anti-Aβ (6E10, SIG-39320; Covance, USA) antibodies. Membranes were washed with TBST buffer (150 mM NaCl, 10 Mm Tris-HCl, and 0.05% Tween-20, pH 8.0) and probed with horseradish peroxidase (HRP) conjugated goat anti-mouse IgG and goat anti-rabbit IgG (12–349, 12-348; Merck Millipore, Germany). Protein signals were visualized using a chemiluminescent HRP substrate ECL detection system (WBKLS0500; Merck Millipore, Germany) and quantified by a luminescence imaging system (LAS-4000; Fujifilm, Japan).

### MTT assay

SHSY-5Y cells reaching 75% confluence were incubated with different concentrations of CSP or un-nanosized curcuminoid for 4 hours at 37°C, and then treated with 5 μM oligomeric Aβ was added at a final concentration of 5 μM. After 48 hours incubation, medium were removed and 10 μL of 3-(4, 5-Dimethylthiazol-2-yl)-2, 5-diphenyltetrazolium bromide (MTT, 10 mg/mL) solution were added for 4 hours incubation. Cells were then lysed with 100 μl of lysis buffer (10% SDS and 20 mM HCl) at 37°C overnight. Cell survival was determined according to the optical density at 570 nm with the ELISA reader (TECAN Sunrise™ Absorbance Reader, Switzerland).

### Phagocytosis assay

BV_2_ microglial cells were seeded in 24-well plates at a density of approximately 8×10^4^ cells in each well. BV_2_ cells were incubated in the presence or absence of 2.2 μM CSP for 1 hour and then incubated with 0.001% fluorescent microspheres beads (F8821; Molecular Probes, USA) coated with fetal calf serum for 3 hours incubation at 37°C. Cells were washed with PBS for 3 times and then stained with Iba1 antibody. The cells and beads were visualized using fluorescence microscopy. The average number of ingested beads per cells was determined as phagocytosis index.

For Aβ clearance, BV2 microglia cells were seeded at a density of 2×10^5^ cells/well on poly-D-lysine coated coverslips. Attached microglia were treated with 1 μM oAβ with 0 or 2.2 μM CSP for 24 hours. The levels of Aβ remaining in the media were determined by Aβ42 ELISA.

### Quantitative real-time PCR (Q-PCR)

The RNA from oAβ and CSP treated microglia were purified using TRI reagent (T9424, Sigma, MO, USA), and then immediately reverse transcribed into cDNA by MMLV high-performance reverse transcriptase (RT80125K, Epicentre, WI, USA). The mRNA expression levels were analyzed by using primers mixed with SYBR Green PCR Master Mix (10476600, Roche, Penzberg, Germany). A StepOnePlus Real-Time PCR System (Applied Biosystem, ABI, MA, USA) was used to monitor the changes of fluorescence intensity from PCR products. GAPDH was used as internal control. Primer sequence are: IL-4 F: 5’GAC GCC ATG CAC GGA GAT3’, R: 5’TCT CTG TGG TGT TCT TCG TTG CT3’; YM1 F: 5’TTC TGG TGA AGG AAA TGC GTA AA3’, R: 5’GCA GCC TTG GAA TGT CTT TCT C3’; GAPDH F: 5’GCA TCC ACT GGT GCT GCC3’; R: 5’TCA TCA TAC TTG GCA GGT TTC3’. The data were analyzed using StepOne software version 2.0.

### Statistical analysis

Statistical analyses were performed with GraphPad Prism (Version 5.0; GraphPad, USA). Differences among multiple means were assessed by one-way, two-way ANOVA, followed by Bonferroni's post-hoc test or Tukey's multiple comparison test. Differences between two means were assessed by paired or unpaired *t* test. The threshold for significance was defined as p< 0.05. All data are presented as mean ±SEM.

## SUPPLEMENTARY MATERIALS TABLES


