# Supplementary material for: Curcuminoid submicron particle ameliorates cognitive deficits and decreases amyloid pathology in Alzheimer’s disease mouse model
Source: Oncotarget. 2018 Jan 31;9(12):10681–97. doi: 10.18632/oncotarget.24369 (PMC5828200; doi:10.18632/oncotarget.24369)
Supplement: Supplementary file 1 [file oncotarget-09-10681-s001.pdf]

Curcuminoid submicron particle ameliorates cognitive deficits and decreases amyloid pathology in Alzheimer's disease mouse model

SUPPLEMENTARY MATERIALS

Supplementary Table 1: The impact of curcuminoid submicron particle (CSP) on micronucleation in male ICR mice in micronucleus assay

	Dose (g/kg)	Micronucleated erythrocytes (%)			Micronucleated reticulocytes (%)		
Negative control (Sterile water)	0	0.53	±	0.14	1.72	±	0.57
Positive control (Cyclophosphamide)	0.10	6.98	±	2.14*	0.20	±	0.18*
Low dose	0.03	0.50	±	0.23	1.72	±	0.42
Moderate dose	0.30	0.61	±	0.21	2.41	±	0.94
High dose	3.00	0.57	±	0.18	2.27	±	1.44

n = 5. Data are presented as mean ± SEM. Results were analyzed by two-way ANOVA ($^{*}p < 0.05$ vs. negative control, Dunnett's *t*-test, 2-sides).

Supplementary Table 2: Mortality of each group in the 28-day and 90-day oral toxicity study of curcuminoid submicron particle (CSP)

	28-day				90-day			
Dose (g/kg/day)	0	0.1	0.5	1.0	0	0.1	0.5	1.0
**Female**								
Number of deaths/total number	0/6	0/6	0/6	0/6	0/8	0/8	0/8	0/8
Mortality rate (%)	0	0	0	0	0	0	0	0
**Male**								
Number of deaths/total number	0/6	0/6	0/6	0/6	0/8	0/8	0/8	0/8
Mortality rate (%)	0	0	0	0	0	0	0	0

n = 6 each group for 28-day study; n= 8 each group for 90-day study

Supplementary Table 3: Organ weight of each group in the 28-day oral toxicity study of curcuminoid submicron particle (CSP)

Organ	Average weight(g)			
	dose (g/kg/day)			
	0	0.1	0.5	1.0
**Female**				
Liver	8.04±0.60	7.85±1.00	8.45±0.55	8.51±0.77
Spleen	0.53±0.07	0.55±0.06	0.56±0.04	0.48±0.05
Kidney	1.73±0.11	1.81±0.21	1.86±0.07	1.72±0.09
Adrenal gland	0.07±0.01	0.08±0.02	0.07±0.01	0.07±0.02
ovary	0.14±0.02	0.14±0.01	0.15±0.02	0.17±0.02*
**Male**				
Liver	11.25±1.77	11.72±2.43	12.32±1.96	10.96±1.02
Spleen	0.67±0.15	0.67±0.08	0.79±0.13	0.63±0.10
Kidney	2.80±0.41	2.79±0.45	2.92±0.25	2.52±0.13
Adrenal gland	0.06±0.01	0.06±0.02	0.07±0.02	0.06±0.01
Testis	3.03±0.20	2.91±0.12	3.11±0.26	2.76±0.56

n = 6. Data are presented as mean ± SEM. Results were analyzed by two-way ANOVA (*, $p < 0.05$ vs. 0 g/kg/day control, Dunnett's *t*-test, 2-sides).

Supplementary Table 4: Organ weight of each group in the 90-day oral toxicity study of curcuminoid submicron particle (CSP)

Organs	Average weight(g)			
	dose (g/kg bw/day)			
	0	0.1	0.5	1.0
**Female**				
Brain	1.92±0.03	1.91±0.05	1.86±0.08	1.86±0.05
Thymus	0.37±0.04	0.30±0.02	0.31±0.02	0.33±0.05
Liver	6.96±0.25	7.31±0.28	7.46±0.34	7.04±0.35
Spleen	0.50±0.02	0.49±0.06	0.53±0.03	0.47±0.03
Kidney	1.65±0.07	1.73±0.05	1.53±0.04	1.51±0.04
Adrenal gland	0.07±0.01	0.07±0.01	0.06±0.01	0.05±0.01
Ovary	0.11±0.01	0.11±0.01	0.13±0.01	0.12±0.01
**Male**				
Brain	1.98±0.06	1.92±0.06	1.99±0.05	1.82±0.04
Thymus	0.42±0.05	0.46±0.03	0.40±0.05	0.40±0.03
Liver	12.40±0.38	13.54±0.73	14.26±0.72	12.54±0.62
Spleen	0.78±0.02	0.79±0.03	0.83±0.05	0.77±0.02
Kidney	2.91±0.07	2.92±0.17	3.11±0.09	3.17±0.20
Adrenal gland	0.06±0.01	0.06±0.01	0.07±0.01	0.06±0.01
Testis	3.49±0.06	3.40±0.11	3.45±0.15	3.21±0.34

n = 8. Data are presented as mean ± SEM. Results were analyzed by two-way ANOVA ($p > 0.05$, Dunnett's *t*-test, 2-sides).
